# Advanced Application of Porcine Intramuscular Adipocytes for Evaluating Anti-Adipogenic and Anti-Inflammatory Activities of Immunobiotics

**DOI:** 10.1371/journal.pone.0119644

**Published:** 2015-03-19

**Authors:** Masahiko Suzuki, Asuka Tada, Paulraj Kanmani, Hitoshi Watanabe, Hisashi Aso, Yoshihito Suda, Tomonori Nochi, Kenji Miyazawa, Kazutoyo Yoda, Fang He, Masataka Hosoda, Tadao Saito, Julio Villena, Haruki Kitazawa

**Affiliations:** 1 Food and Feed Immunology Group, Graduate School of Agricultural Science, Tohoku University, Sendai 981-8555, Japan; 2 Cell Biology Laboratory, Graduate School of Agricultural Science, Tohoku University, Sendai 981-8555, Japan; 3 Laboratory of Immunobiotechnology, Reference Centre for Lactobacilli (CERELA-CONICET), Tucuman, Argentina; 4 Department of Food, Agriculture and Environment, Miyagi University, Sendai 982-0215, Japan; 5 Technical Research Laboratory, Takanashi Milk Products Co., Ltd, Yokohama Kanagawa, 241-0023, Japan; Charité, Campus Benjamin Franklin, GERMANY

## Abstract

We previously established a clonal porcine intramuscular preadipocyte (PIP) line and we were able to establish a protocol to obtain functional mature adipocytes from PIP cells. We hypothesized that both PIP cells and mature adipocytes are likely to be useful *in vitro* tools for increasing our understanding of immunobiology of adipose tissue, and for the selection and study of immunoregulatory probiotics (immunobiotics) able to modulate adipocytes immune responses. In this study, we investigated the immunobiology of PIP cells and mature adipocytes in relation to their response to TNF-α stimulation. In addition, we evaluated the possibility that immunobiotic microorganisms modify adipogenesis and immune functions of porcine adipose tissue through Peyer’s patches (PPs) immune-competent cells. We treated the porcine PPs immune cells with different probiotic strains; and we evaluated the effect of conditioned media from probiotic-stimulated immune cells in PIP cells and mature adipocytes. The *Lactobacillus* GG and *L*. *gasseri* TMC0356 showed remarkable effects, and were able to significantly reduce the expression of pro-inflammatory factors and negative regulators (A20, Bcl-3, and MKP-1) in adipocytes challenged with TNF-α. The results of this study demonstrated that the evaluation of IL-6, and MCP-1 production, and A20 and Bcl-3 down-regulation in TNF-α-challenged adipocytes could function as biomarkers to screen and select potential immunobiotic strains. Taking into consideration that several *in vivo* and *in vitro* studies clearly demonstrated the beneficial effects of *Lactobacillus* GG and *L*. *gasseri* TMC0356 in adipose inflammation, the results presented in this work indicate that the PIP cells and porcine adipocytes could be used for the screening and the selection of new immunobiotic strains with the potential to functionally modulate adipose inflammation when orally administered.

## Introduction

The incidence of obesity has risen continuously over the last decades, and the associated medical and economic costs to society are substantial. Obesity is often accompanied with metabolic syndromes and increased risk for development of various life threatening health complications such as inflammation, type 2 diabetes, cardiovascular diseases, hypercholesterolaemia, cancer, hypertension, and respiratory problems [1–3]. Adipose tissue inflammation is proposed as a central factor connecting obesity with its metabolic and vascular complications. In fact, obesity-induced inflammation exerts profound effects on metabolic pathways, playing one of the central roles in the development of insulin resistance [4, 5].

Adipose tissue is considered as a major storage compartment for lipid accumulation in mammals. This tissue is not homogenous, it contains various cellular components such as preadipocytes, mature adipocytes, fibroblasts, macrophages and endothelial cells; capable of differentiate into other cell types; being mature adipocytes the dominant cell type [6, 7]. Preadipocytes are able to proliferate and differentiate into lipid-laden or insulin responsive mature adipocyte, determining the number of fat cells that will exist throughout the entire lifespan [7]. Adipose tissue is constituted by remarkable active endocrine cells that secrets a number of adipokines: adiponectins, leptin, visfatin, resistin, serum amyloid A3, omentin and RBP4, and inflammatory cytokines: tumor necrosis factor (TNF)-α, interleukin (IL)-6, IL-1, IL-10, monocyte chemoattractant protein (MCP)-1 and interferon (IFN)-γ. Those factors play pivotal roles in the regulation of various physiological and pathological processes in which adipose tissue is involved [6, 8].

TNF-α is a multifactorial regulatory cytokine, which has been implicated as mediator in induction of insulin resistance and adipose tissue inflammation [9–11]. This cytokine is elevated in the adipose tissues of obese mice and humans [10]. TNF-α is believed to regulate adipocyte metabolism and immune activities by modulating glucose and fatty acid metabolism, inflammatory genes expression, transcriptional regulation and hormone receptor signaling [8, 9]. Studies reported that administration of TNF-α increased the glucose homeostasis and insulin resistance in animals and humans [12, 13]. Moreover, some reports described that deletion or lacking of TNF-α gene allowed the protection against the development of insulin resistance in obese mice [14]. Some human studies demonstrated that treatment of obese subjects with TNF-α antagonists is able to beneficially modulate glucose metabolism and inflammation [15, 16]. Then, regulation of TNF-α signaling pathway in adipocytes could be one strategy to control undesirable metabolic and immune effects of obesity.

Healthy food and life style habits have been recommended to avoid obesity-associated diseases. Thus, finding natural and safe dietary supplements able to modulate adipocytes function in general, and TNF-α signaling pathway in particular, would be of value to prevent obesity associated diseases. Probiotics are one of the functionally proved effective and safe dietary supplements to restrain body obesity and insulin resistance. Some scientific *in vivo* studies reported that probiotics supplementation reduced high fat diet induced obesity, decreased insulin resistance, and beneficially modulated inflammatory response in rodent models [17, 18]. High-fat diet induced obese mice treated with *Lactobacillus rhamnosus* GG improved insulin sensitivity and reduced lipid accumulation. Those effects were associated to reductions of glucose transporter (GLUT4) expression and secretion of adiponectin [17]. Recently, it was reported that the administration of *L*. *coryniformis* CECT5711 to obese mice induced marked changes in microbiota composition, reduced the metabolic endotoxaemia as it decreased lipopolysaccharide (LPS) and TNF-α plasma levels, and improved endothelial dysfunction and vascular oxidative stress [18].

In a previous work, we demonstrated that the murine macrophage-like cell line J774.1 treated with *L*. *rhamnosus* GG or *L*. *gasseri* TMC0356 improved the production of IL-6 and IL-12 [19]. The conditioned medium from lactobacilli-cultured J774.1 cells transferred to the preadipocyte cell line 3T3-L1 significantly suppressed lipid accumulation and decreased peroxisome proliferator activated receptor γ (PPAR-γ) [19]. Similarly, Park et al. [20] showed that exposure of 3T3-L1 adipocytes to *L*. *brevis* OPK-3 inhibited intracellular lipid accumulation by decreasing PPAR-γ and CCAAT/enhancer binding protein α. Development of established preadipocyte cell lines, such as 3T3-L1, greatly facilitated the study of molecular mechanisms of adipocyte differentiation and inflammatory response under defined conditions. These cell lines are derived from mouse, and preadipocyte cell lines of other species have not yet been maintained in culture long enough to study differentiation or immune responses. Some porcine preadipocytes cell lines have been developed which maintain a normal phenotype without transforming spontaneously even after long-term maintenance in culture [21, 22]. In this regard, we have established a clonal porcine intramuscular preadipocyte (PIP) line from the *Musculus longissimus thoractis* of a Duroc pig. Moreover, we used this cell line for the investigation of adipogenic differentiation and we were able to establish a protocol to obtain functional mature adipocytes from PIP cells [21]. Both PIP cells and mature adipocytes are likely to be useful *in vitro* tools for increasing our understanding of adipogenesis and immunobiology of adipose tissue.

In this study, we investigated the immunobiology of PIP cells and mature adipocytes in relation to their response to TNF-α stimulation. In addition, we investigated the possibility of immunoregulatory probiotics that modify adipogenesis and immune functions of porcine adipose tissue through Peyer´s patches (PPs) immune-competent cells. We treated the porcine PPs immune cells with different immunobiotic strains [23]; and we evaluated the effect of conditioned media from immunobiotic-stimulated immune cells in porcine preadipocytes (PIP cells) and mature adipocytes.

## Materials and Methods

### Cell culture and induction of adipogenesis

Porcine intramuscular preadipocyte (PIP) cells, which are derived from marbling muscle tissue of the *musculus longissimus thoracis* from female Duroc pig [21], were maintained in Dulbecco’s modified Eagle medium (DMEM, Gibco, Paisley, Scotland, UK) supplemented with 10% fetal calf serum (FCS), 100 mg/ml penicillin, and 100 U/ml streptomycin as a growth medium. PIP cells were plated at density of 2.5×10^4^/cm^2^ in 6-well cell culture plates (BD Falcon, Tokyo, Japan) and incubated at 37°C in a humidified atmosphere of 5% CO_2_. The 4-day post-confluent PIP cells were fed with differentiation medium for another 4 more days to induce differentiated adipocyte. The differentiation medium was DMEM containing 10% FBS, 50 ng/ml insulin (swine, Sigma), 0.25 μM dexamethasone (Sigma), 2 mM octanoate (Wako), 200 μM oleate (Ardorich, Milwaukee, WI, USA), 100 U/ml penicillin, and 100 μg/ml streptomycin. The medium was changed every day. All experiments were performed between the 26^th^ and 35^th^ passages of PIP cells.

### Oil red O and hematoxylin stain

Accumulation of intracellular lipid droplets in PIP cells and differentiated adipocytes were quantified after staining with Oil red O. Cells were washed twice with phosphate-buffered saline (PBS) and fixed on ice with 10% formaline for 30 minutes, and then cells were dissolved in isopropanol for 1 minute and stained with Oil red O (SIGMA-ALDRICH, Tokyo, Japan) for 30 minute at 37°C. Thereafter, cells were washed twice with deionized water (DW) and stained with Mayer's Hematoxylin Solution (MERCK, Darmstadt, Germany). The stained cells were then washed with DW and immersed in aqueous ammonia. Then, the cells were dried to observe and photograph lipid droplet accumulation under the biological microscope (OLYMPUS IX70). Photographs of cells were also imaged using Image J (National Institutes of Health, Bethesda, MD) and measured Oil red O stained area and evaluated area per cell.

### Quantitative Real-time Polymerase Chain Reaction (RT-PCR)

The expression of pattern recognition receptors (PRRs), cytokines and chemokines (IL-1α, IL-1β, IL-6, IL-8 and MCP-1) and negative regulators (MKP-1, A20 and Bcl-3) in both PIP and differentiated adipocyte were evaluated by RT-PCR under non-inflammatory conditions and after the challenge with different concentration of TNF-α (2.5, 25, 250 ng/ml) for 24 h. Total RNA was isolated from each cell sample using TRIzol reagent (Invitrogen) and cDNAs were synthesized using a Quantitect reverse transcription (RT) kit (Qiagen, Tokyo, Japan) according to the manufacturer’s recommendations. Real-time quantitative PCR was carried out using a 7300 real-time PCR system (Applied Biosystems, Warrington, UK) and the Platinum SYBR green qPCR SuperMix uracil-DNA glycosylase with 6-carboxyl-X-rhodamine (Invitrogen). The primer sequences for TNF receptors are as follows: TNFR1 (sense 5’-GGTCCATTTGCTGCACGAA-3’, antisense 5’-GGGCCCAGACAGTCATTGTG-3’), TNFR2, (sense 5’- GCTCCACGAGGGACACAGA-3’, antisense 5’- AAGGGACCTGCTCATCTTTGG-3’), Nod1 (Sense 5’-CTGTCGTCAACACCGATCCA-3’), antisense 5’-CCAGTTGGTGACGCAGCTT-3, and Nod2 (sense 5’-GAGCGCATCCTCTTAACTTTCG-3’, antisense 5’- ACGCTCGTGATCCGTGAAC-3’). The primers used for the analysis of PRRs, cytokines, chemokines and TLR negative regulators were described previously [24, 25]. The PCR cycling conditions were 5 minutes at 50°C, followed by 5 minutes at 95°C, and then 40 cycles of 15 seconds at 95°C, 30 seconds at 60°C, and 30 seconds at 72°C. The volume of reaction mixtures was 10 μl, which contains 2.5 μl of cDNA and 7.5 μl of master mix included the sense and antisense primers. Expression of β-actin in each sample was assessed, and the β-actin data was used as an internal control to normalize differences between samples and to calculate relative expression levels.

### Preparation of lactic acid bacterial (LAB) strains

*Lactobacillus gasseri* TMC0356, *L*. *rhamnosus* GG, *L*. *rhamnosus* LA-2, *L*. *paracasei* TMC0409, *Streptococcus thermophilus* TMC1543, *Bifidobacterium bifidum* TMC3108, and *B*. *bifidum* TMC3115 were used in this study, which were kindly gifted by Takanashi milk product company, Japan. The lactobacilli and bifidobacterial strains were grown in deMan-Rogosa-Sharp (MRS, Difco, Detroit, MI, USA) medium for 16 hours at 37°C. *S*. *thermophilus* were cultured in Elliker medium (Difco) at 37°C for 16 hours. All these bacterial strains were washed with PBS and sterilized by heating at 60°C for 30 minutes. After washing twice with PBS, the cells resuspended in DMEM (10% FCS, 1% streptomycin/penicillin) and adjusted cell concentration with 2.5×10^9^ cells/ml.

### Immunomodulatory activity of LAB in PIP cells and differentiated adipocytes

For the evaluation of anti-inflammatory and anti-adipogenic activity of LAB strains, we performed several *in vitro* steps to simulate LAB-immune cells-adipocytes interaction. For this purpose, we first stimulated mononuclear cells isolated from porcine Peyer’s patches with the different LAB strains. The immunocompetent cells isolated from porcine Peyer’s patches according to our previous method [26] were placed in a 6-well cell culture plate (BD Falcon, 1 x 10^6^ cells/well). Then the cells were stimulated with LAB (5 x 10^7^ cells /well) at 5% CO_2_, 37°C, for 24 hours, in a complete RPMI 1640 medium (Sigma) supplemented with 2% fetal bovine serum (FCS). After stimulation, cell free supernatant (CFS) was collected for further experiments. Both PIP and differentiated adipocyte were pre-stimulated for 48 hours with CFS. Then, cells were post-stimulated with TNF-α for 3, 6, and 12 hours at 37°C, 5% CO_2_. Accumulation of intracellular lipid droplets were quantified after staining with Oil red O. In addition, the expression of immune receptors, cytokines and negative regulators was quantified by RT-PCR.

### Statistical Analysis

Analysis of variance (One-way ANOVA) was performed by GLM procedure of SAS programs (Version 9.1). The mean comparisons between the PIP cells and adipocyte copy numbers, and mean comparisons among the values of relative indices, and the normalized fold expressions were carried out using tukey-kramer’s method. Relative indices were calculated as the ratio of fat deposition in adipocyte cells; and the values are normalized by common logarithmic transformation and confirmed as approximate values included significantly into normal distribution. Then, they were adjusted similarly to the mean of control groups. The mRNA expression in the stimulated time showed as the ratio of cytokine expressions induced by TNF-α, which was normalized by common logarithmic transformation.

## Results

### Immune characterization of PIP cells and differentiated adipocytes

**Fig 1:**
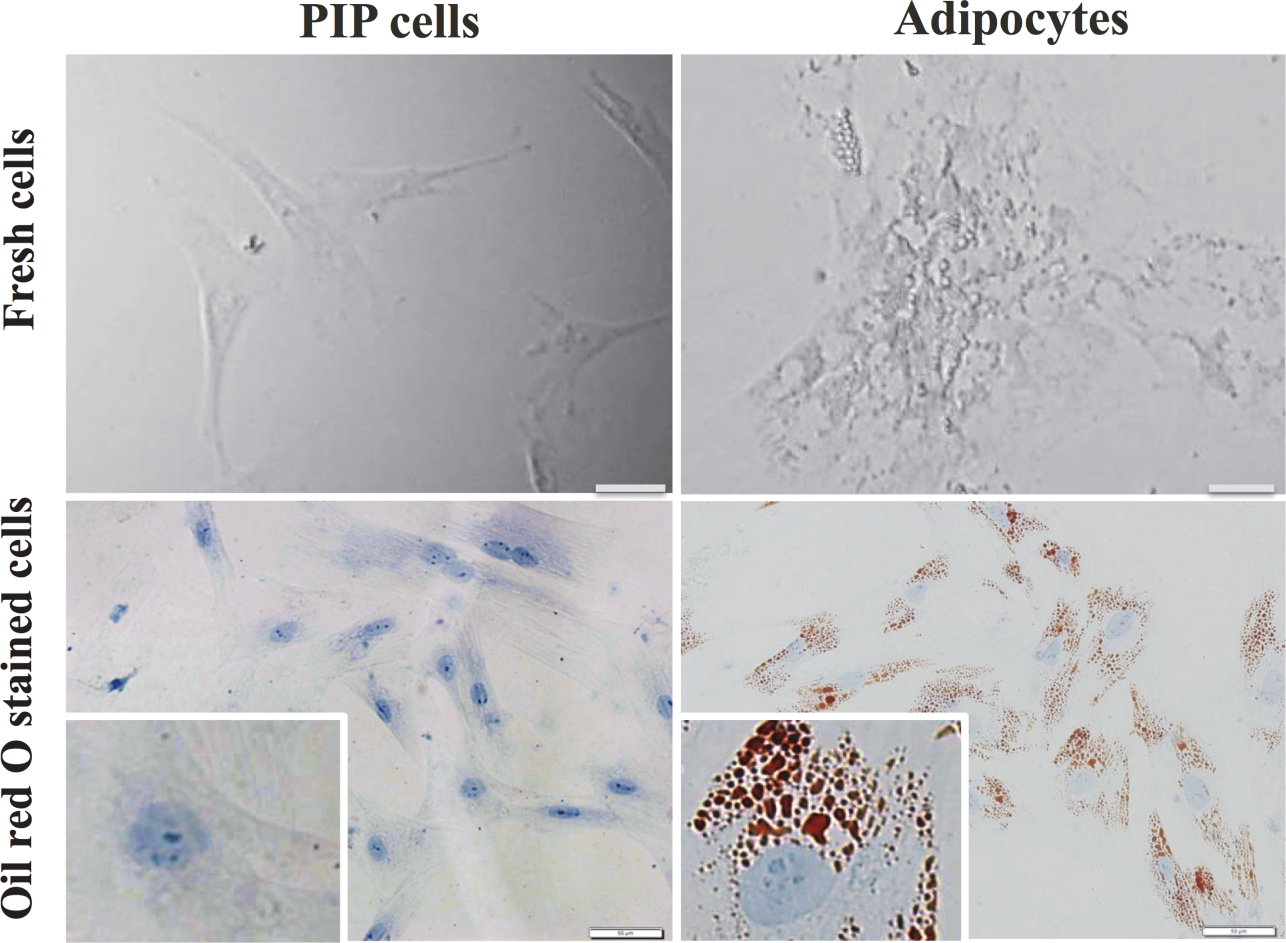
Photographic pictures of PIP and adipocyte cells. The PIP cells were grown at 37°C for 4 days, and subsequently fed with differentiation medium for another 4 days to differentiate into adipocyte cells. Before and after adipocyte differentiation, the cells were stained with Oil red O stain. Presence of red and blue color indicates accumulation of lipid droplets and nucleus in the adipocyte cells at 8 day.

We first aimed to morphologically characterize PIP cells and differentiated porcine adipocytes. Fresh cells as well as cells stained with Oil red O were observed. As shown in Fig. 1, remarkable differences were observed between PIP cells and adipocytes. PIP cells were found to be small cells with a clear nucleus at the center surrounded by abundant cytoplasm. On the contrary, differentiated adipocytes were observed as bigger cells than PIP cells and showed lipid droplets that pushed nucleus to the periphery.

**Fig 2:**
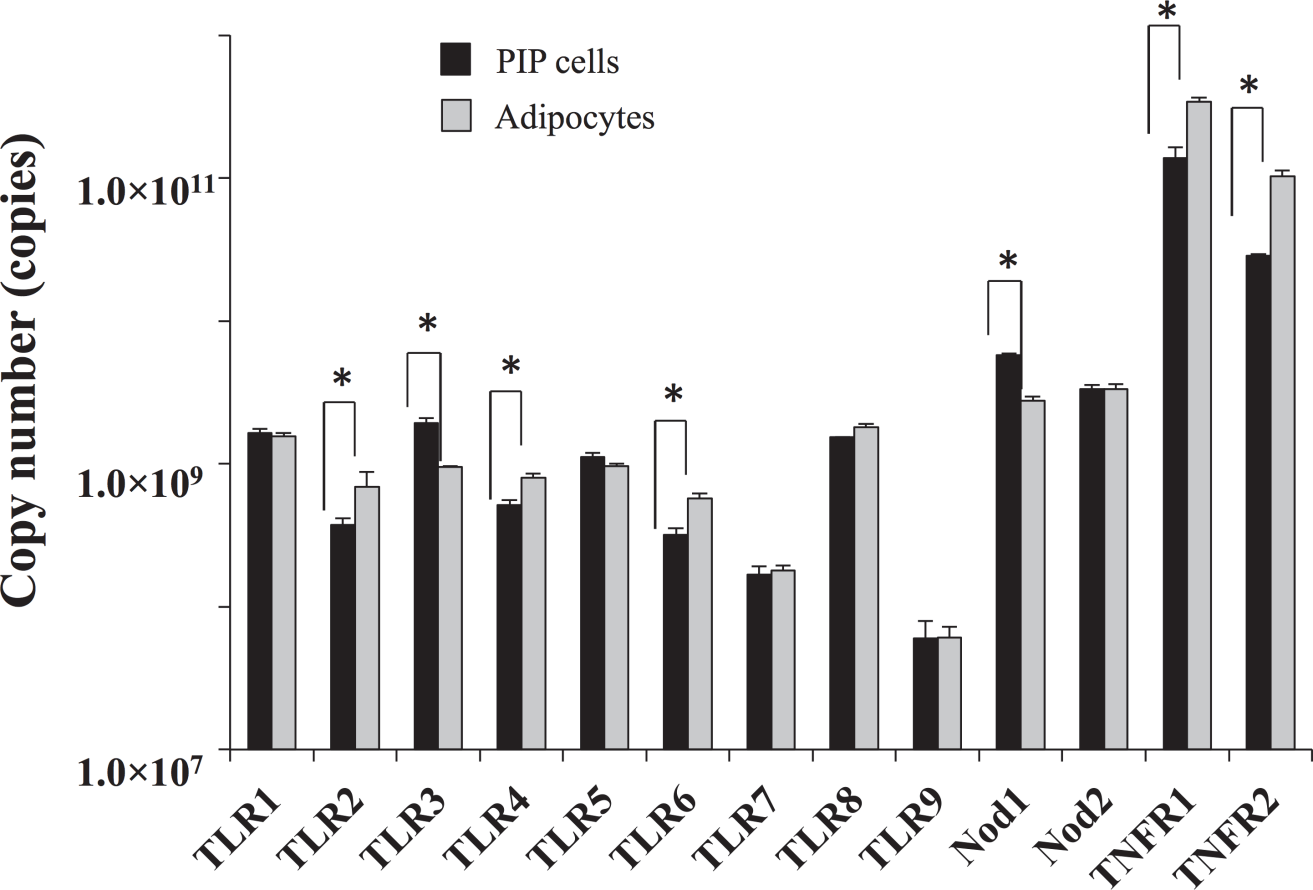
Expression of pattern of PPRs (TLR1–9, NOD 1, 2) and TNFR1, 2 in PIP, and differentiated adipocyte cells. Copy number of mRNA in 25 ng of cDNA prepared from PIP and adipocyte cells were calculated. Three independent experiments were conducted for each case and the average values (mean ± S.D) were represented. The presence of stars (*) indicates statistical differences with significant levels of p<0.05.

In addition, expression of various PRRs (TLR1–9, NOD-1 and 2) and TNFR-1 and 2 were analyzed in PIP cells and differentiated adipocytes (Fig. 2). Both PIP cells and adipocytes expressed TLR1–9, NOD-1, NOD-2, TNFR-1 and TNFR-2. However, TNFR-1 and TNFR-2 were highly expressed when compared with other receptors in both PIP cells and differentiated adipocytes. We also observed that PIP cells showed a higher expression of NOD-1 and TLR3 when compared with adipocytes (Fig. 2). No significant differences were observed in the expression of TLR1, TLR5, TLR7–9 and NOD-2; while the expression of TLR2, TLR4, TLR6, TNFR-1 and TNFR-2 was higher in adipocytes than in PIP cells (Fig. 2).

### Response of PIP cells and adipocytes to TNF-α stimulation

**Fig 3:**
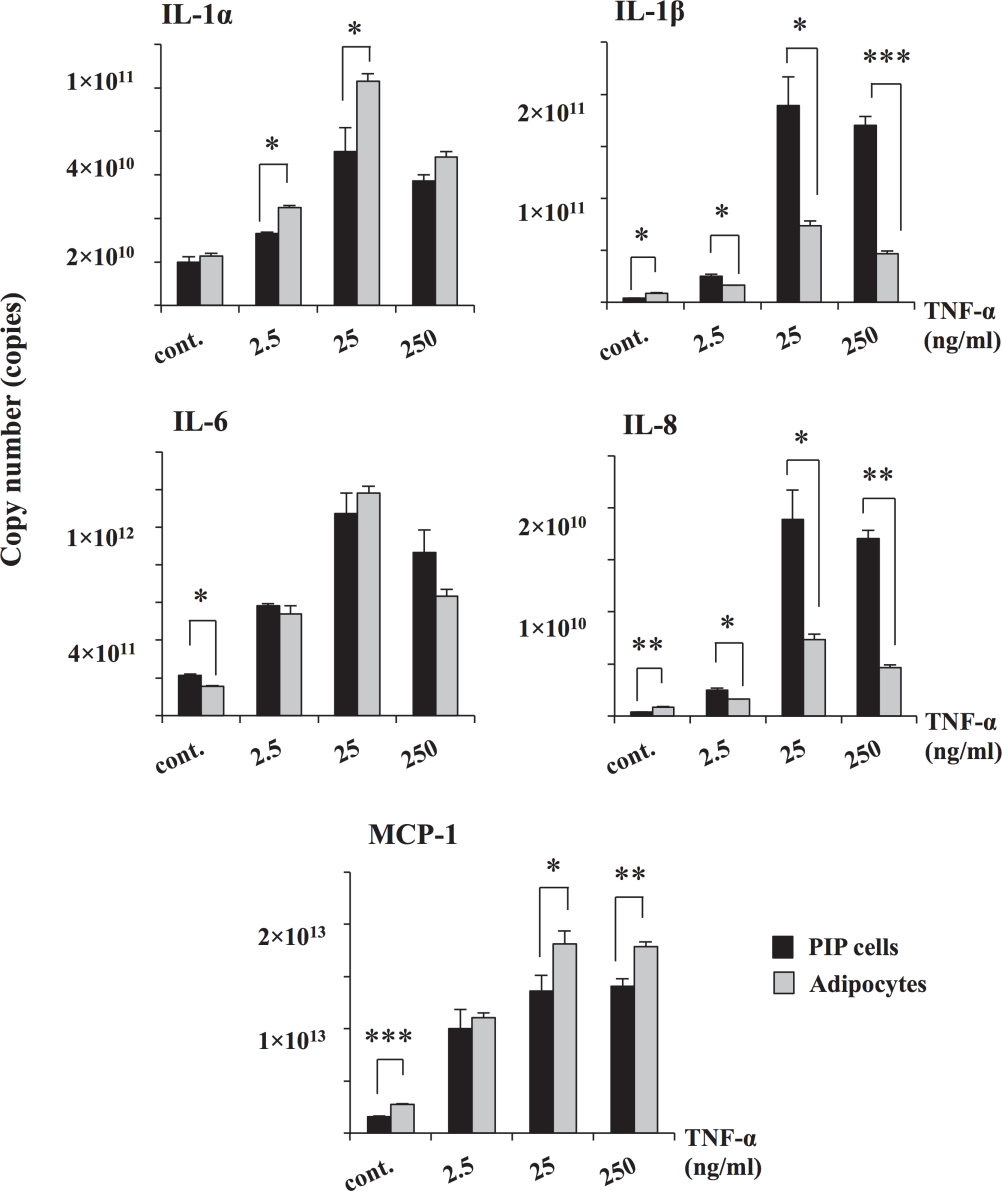
TNF-α induced inflammatory cytokines in PIP and differentiated adipocyte cells. Both PIP cells and adipocytes were incubated with different concentrations of TNF-α (2.5, 25, 250 ng/ml) for 24 hours and the level of pro-inflammatory cytokines (IL-1α, IL-1β, IL-6, IL-8, and MCP-1) were determined. Cells without addition of TNF-α were used as controls. Three independent experiments were conducted for each case and the average values (mean ± S.D) were represented as inflammatory effect of TNF-α. The presence of various stars (*, ** and ***) indicates statistical differences with significant levels of p<0.05, p<0.01 and p<0.001, respectively.

**Fig 4:**
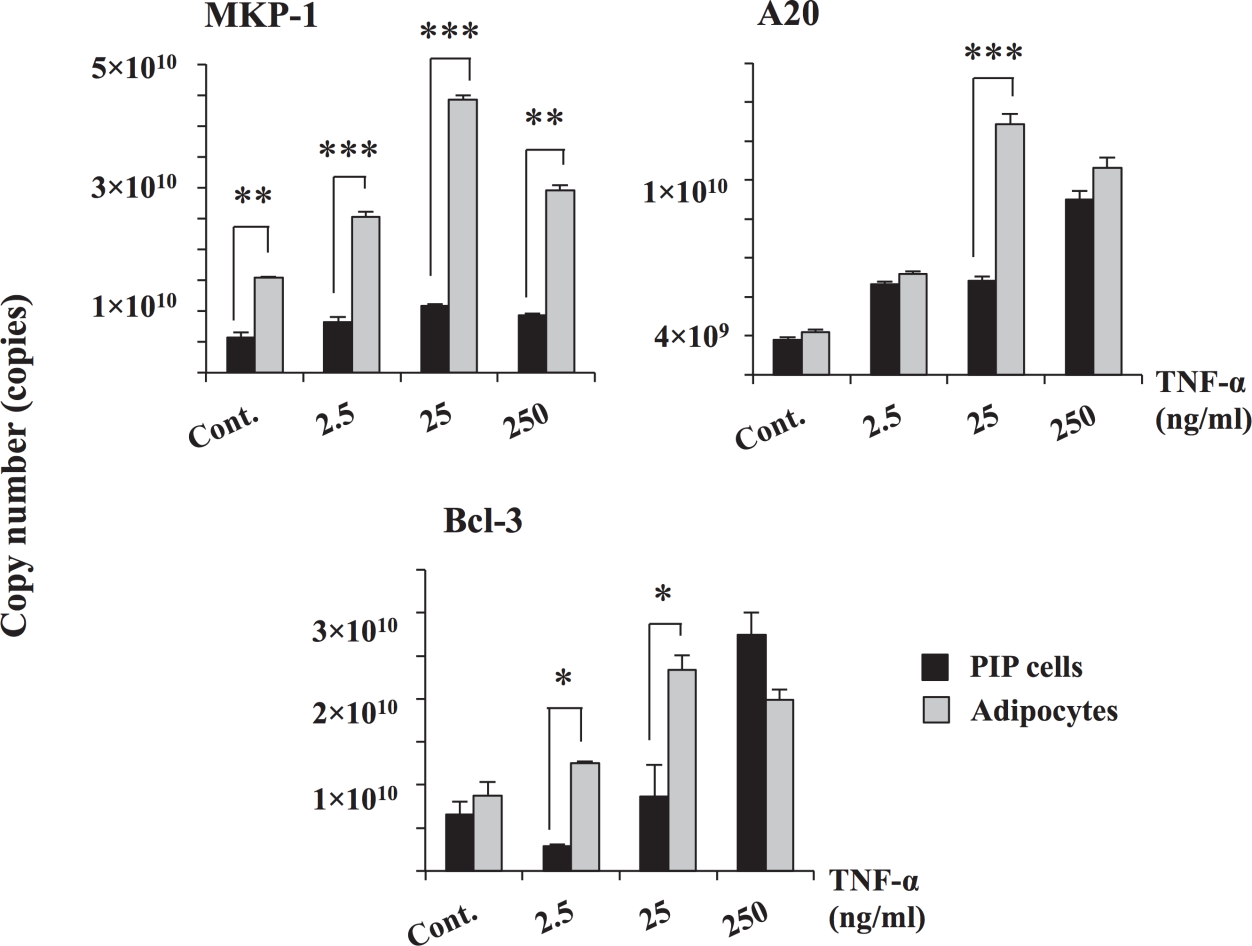
Expression of negative regulators in PIP and differentiated adipocyte cells. Both PIP cells and adipocytes were challenged with different concentrations of TNF-α (2.5, 25, and 250 ng/ml) for 24 hours and the level of negative regulators (MKP-1, A20, and Bcl-3) were determined. Cells without TNF-α stimulation were used as controls. Three independent experiments were conducted for each case and the average values (mean ± S.D) were represented as inflammatory effect of TNF-α. The presence of various stars (*, ** and ***) indicates statistical differences with significant levels of p<0.05, p<0.01 and p<0.001, respectively.

We next aimed to evaluate the effect of TNF-α in the induction of inflammatory cytokines by PIP cells and adipocytes. Fig. 3 shows the expression of cytokines in PIP and adipocyte cells. The expressions of all cytokines were significantly up-regulated when cells were stimulated with TNF-α. For all the pro-inflammatory cytokines and chemokines studied, the maximum expression was observed with concentration of 25 ng/ml of TNF-α. In addition, significant differences in the expressions of cytokines were observed between PIP cells and adipocytes. IL-1β, and IL-8 mRNA levels were significantly higher in PIP cells than in adipocytes after TNF-α stimulation. On the contrary, IL-1α and MCP-1 expressions were higher in adipocytes than in PIP cells (Fig. 3). In particular, the MCP-1 expression was highest in this assay. We also evaluated the effect of TNF-α in the expressions of the negative regulators MKP-1, A20 and Bcl-3 in both PIP cells and differentiated adipocytes (Fig. 4). Challenge of PIP cells with TNF-α significantly increased the expression of A20 and Bcl-3 while no effect was observed in the expression of MKP-1. Similarly, stimulation of differentiated adipocytes with TNF-α increased the expression of A20 and Bcl-3 reaching levels that were significantly higher than those observed in PIP cells (Fig. 4). Moreover, TNF-α increased mRNA levels of MKP-1 in differentiated adipocytes. As observed with cytokines, for negative regulators, higher levels of expression were also observed with 25 ng/ml of TNF-α. Therefore, this concentration of TNF-α was selected for further experiments.

### Evaluation of anti-inflammatory and anti-adipogenic activity of LAB strains

**Fig 5:**
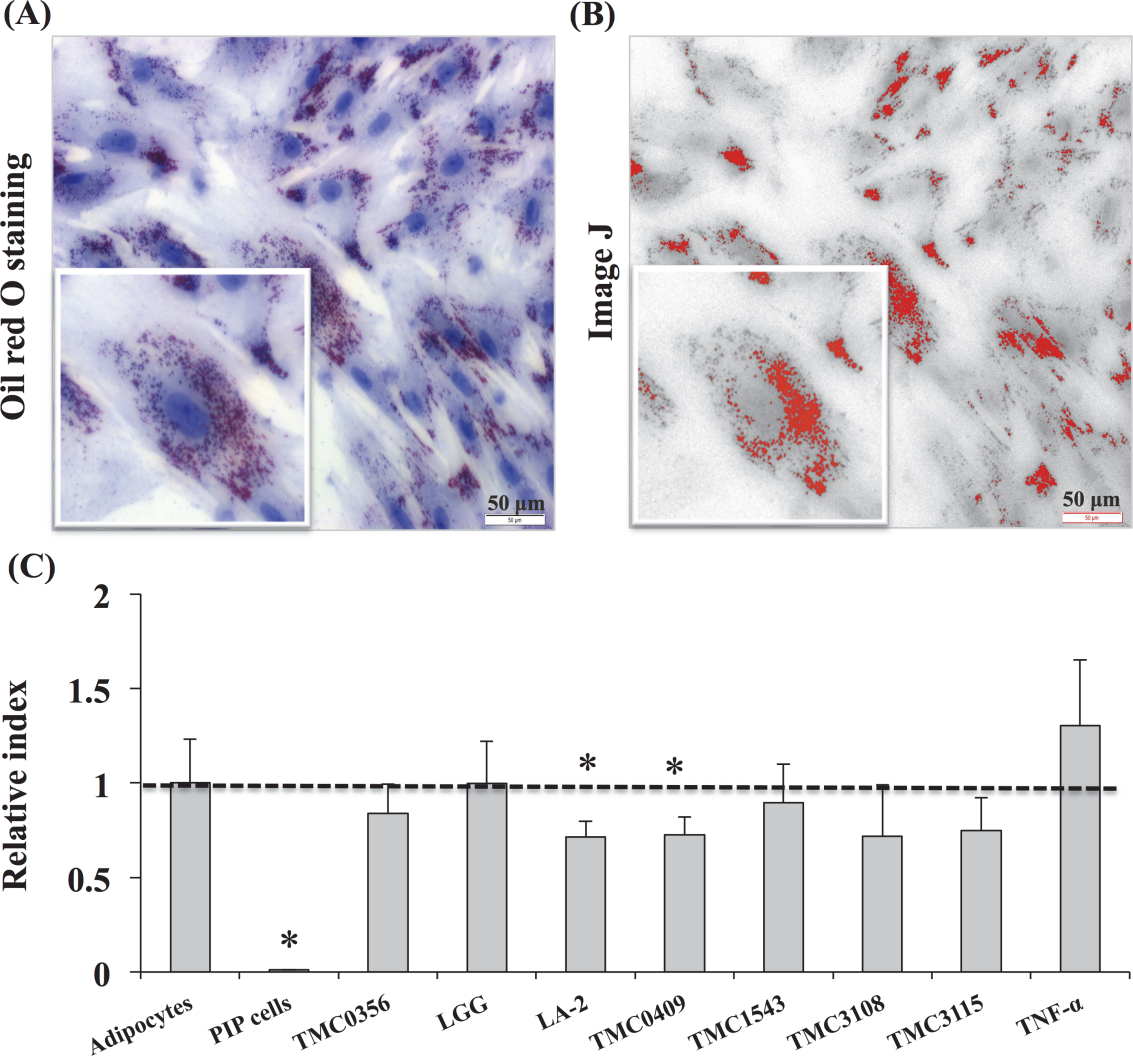
Anti-adipogenic activity of various LABs. The PIP and differentiated adipocytes were incubated with CFS of various LABs treated with PPs at 37°C for 48 hours. Mature adipocyte cells were Oil red O stained and deposition of lipid droplets in the mature adipocytes was quantified by image J. (A) Oil red O stained mature adipocytes. (B) Photograph of image J with lipid accumulation. (C) Anti-adipogenic activity of LABs. Three independent experiments were conducted for each case and the average values (mean ± S.D) were represented as anti-adipogenic effect of LABs. The presence of stars (*) indicates statistical differences with significant levels of p<0.05.

For the evaluation of the anti-adipogenic and immunoregulatory activity of LAB, we performed several *in vitro* steps to simulate LAB-immune cells-adipocytes interaction as described in materials and methods. Then, PIP cells and adipocytes were stimulated with CFS from porcine Peyer’s patche immunocompetent cell cultures stimulated with different LAB strains. PIP cells and adipocytes were pre-stimulated with the different CFS for 48 hours and then challenged with TNF-α for 3, 6, and 12 hours. The effect of LAB on PIP cells differentiation and adipogenesis was investigated by using Oil red O staining (Fig. 5). The deposition of fats in the mature adipocytes was quantified by image J. Deposition of lipids increased with differentiation of PIP cells into mature adipocytes, and fat deposit were significantly increased after stimulation with TNF-α (Fig. 5). In mature adipocytes coming from PIP cells treated with culture free supernatants from LA-2 and TMC0409 strains, significantly lower deposition of lipids was observed when compared to control cells (Fig. 5).

**Fig 6:**
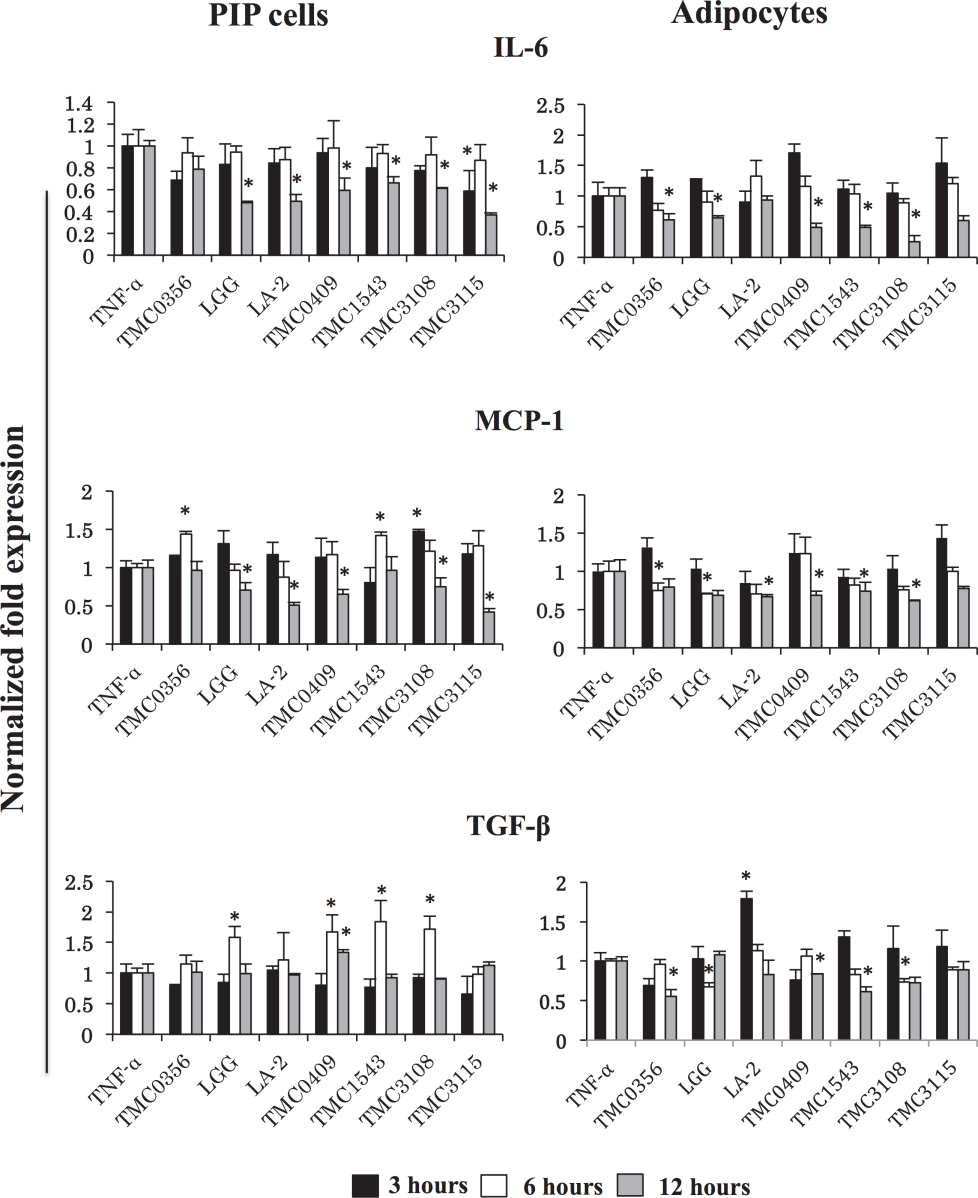
Anti-inflammatory activity of various LABs in PIP cells and differentiated adipocytes. Both PIP cells and differentiated adipocytes were pre-stimulated with CFS of various LABs for 48 h and then post-stimulated with TNF-α for 3, 6, and 12 hours. Cells without any TNF-α stimulation were used as cell controls. The expressions of IL-6, MCP-1, and TGF-β mRNAs were evaluated. Cells treated only with TNF-α were used as TNF-α controls. Three or four independent experiments were conducted for each case and the average values (mean ± S.D) were represented as immunomodulatory effect of LABs. The presence of stars (*) indicates statistical differences with significant levels of P<0.05.

In addition, the mRNA levels of TLR2, TLR4, IL-6, MCP-1, and TGF-β were determined in PIP cells and adipocytes as shown in Fig. 6. As mentioned earlier, TNF-α was capable to induce mRNA expressions of IL-6, MCP-1, TLR2 and TLR4 in both PIP cells and differentiated adipocytes (Fig. 3), while no modification was observed in the expression of TGF-β (data not shown). A strain dependent effect was observed in the levels of IL-6, MCP-1, and TGF-β when PIP cells were pretreated with cell free supernatants. PIP cells pretreated with cell free supernatant from *L*. *rhamnosus* GG, *L*. *paracasei* TMC0409 or *B*. *bifidum* TMC3108 showed increased expression of TGF-β earlier at hour 6 post-TNF-α challenge, while those cells showed reduced levels of IL-6 and MCP-1 at hour 12 (Fig. 6). *L*. *rhamnosus* LA-2 was able to significantly reduce levels of IL-6 and MCP-1 but did not induce changes in TGF-β expression (Fig. 6). *L*. *gasseri* TMC0356 significantly increased MCP-1. In addition, *S*. *thermophilus* TMC1543 reduced IL-6 levels but increased the expression of MCP-1; while *B*. *bifidum* TMC3115 reduced IL-6 and MCP-1 levels (Fig. 6). Interestingly, almost all the studied LAB strains induced similar effects in differentiated adipocytes after TNF-α challenge. *L*. *gasseri* TMC0356, *L*. *rhamnosus* GG, *L*. *paracasei* TMC0409, *S*. *thermophilus* TMC1543 and *B*. *bifidum* TMC3108 significantly reduced levels of IL-6, MCP-1, and TGF-β in TNF-α-challenged porcine adipocytes (Fig. 6). On the contrary, *L*. *rhamnosus* LA-2 significantly increased levels of TGF-β in adipocytes.

**Fig 7:**
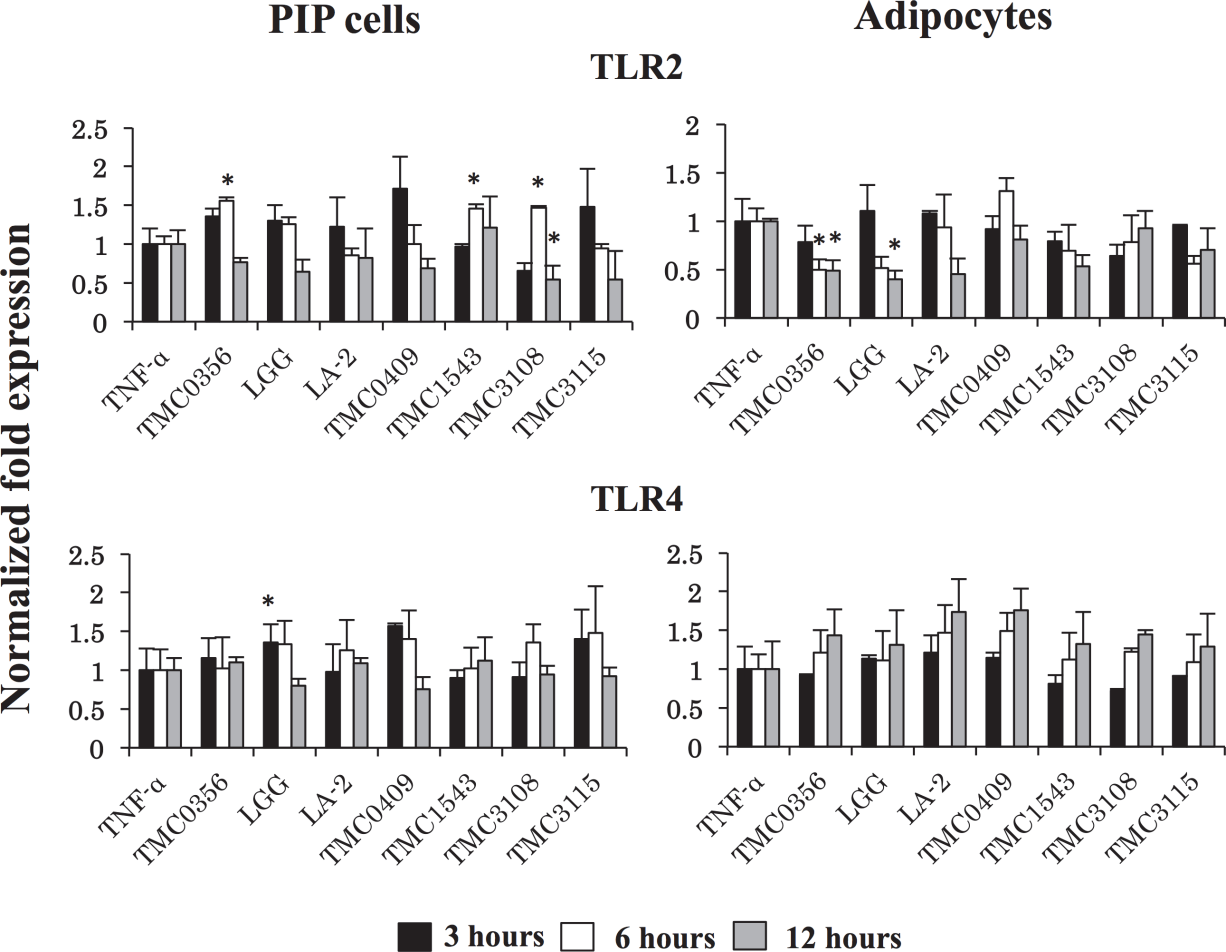
Induction of TLRs expression in PIP cells and differentiated adipocytes. Both PIP cells and differentiated adipocytes were pre-stimulated with CFS of various LABs for 48 h and then post-stimulated with TNF-α for 3, 6, and 12 hours. The expressions of TLR2 and TLR4 were evaluated. Cells treated only with TNF-α were used as TNF-α controls. Three or four independent experiments were conducted for each case and the average values (mean ± S.D) were represented as immunomodulatory effect of LABs. The presence of stars (*) indicates statistical differences with significant levels of P<0.05.

Strain dependent effects were also observed when analyzing TLR2 and TLR4 expressions in PIP cells and adipocytes pretreated with cell free supernatants (Fig. 7). *L*. *gasseri* TMC0356, *S*. *thermophilus* TMC1543 and *B*. *bifidum* TMC3108 significantly increased TLR2 expression in PIP cells, while *L*. *rhamnosus* GG augmented mRNA levels of TLR4. In addition, *L*. *gasseri* TMC0356 and *L*. *rhamnosus* GG reduced TLR2 levels in differentiated adipocytes while no significant changes were observed in the expression of TLR4 in these cells with CFS treatments (Fig. 7).

**Fig 8:**
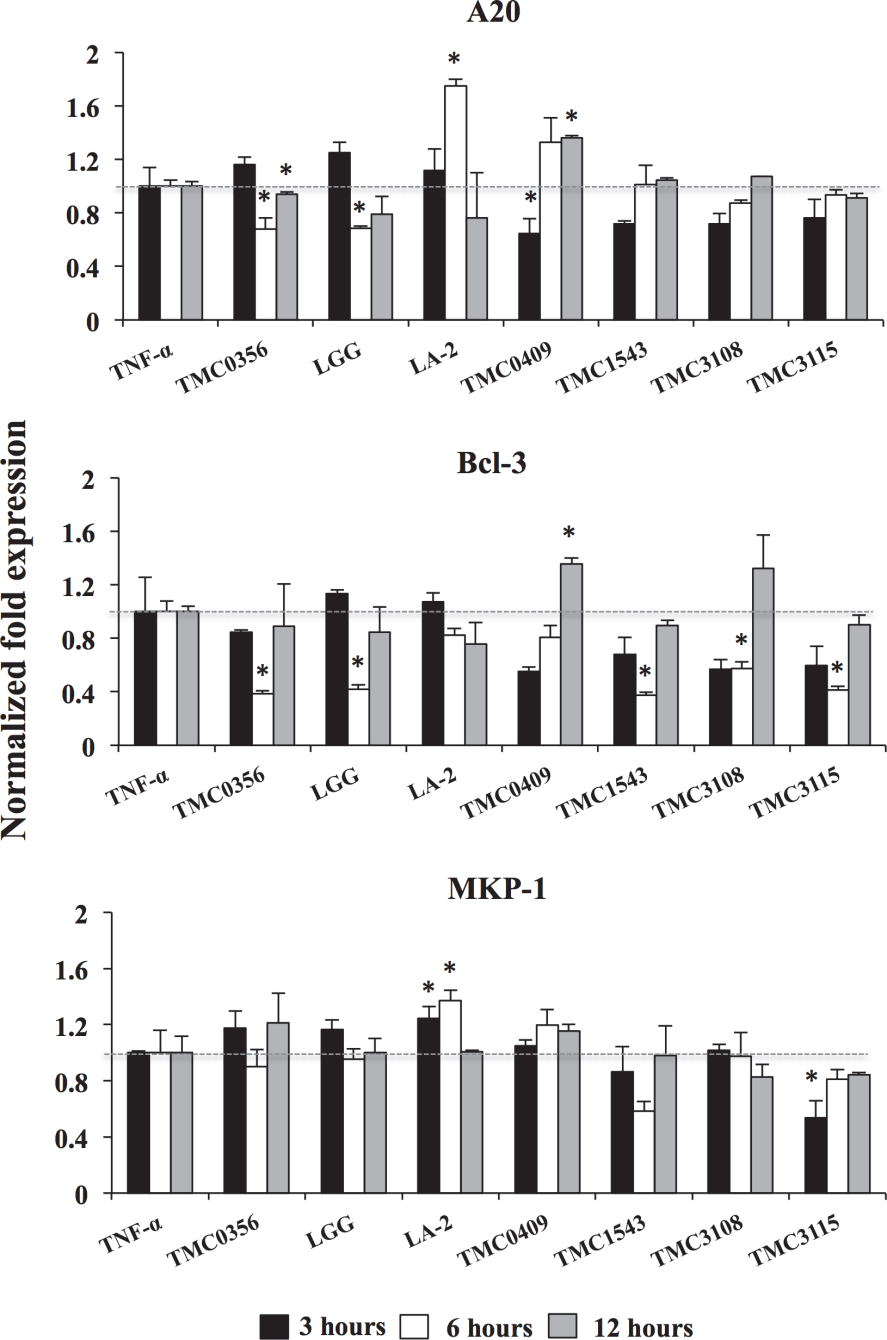
Expression of negative regulators in differentiated adipocytes. PIP cells and differentiated adipocytes were pre-stimulated with CFS of various LABs for 48 h and then post-stimulated with TNF-α for 3, 6, and 12 hours. The expressions of A20, Bcl-3, and MKP-1 were evaluated. Cells treated only with TNF-α were used as TNF-α control. Three or four independent experiments were conducted for each case and the average values (mean ± S.D) were represented as regulatory effect of LABs. The presence of stars (*) indicates statistical differences with significant levels of p<0.05.

Finally we aimed to evaluate whether CFS was able to modulate the expressions of negative regulators after TNF-α challenge in porcine adipocytes (Fig. 8). Again, strain dependent effects were observed. *L*. *gasseri* TMC0356 and *L*. *rhamnosus* GG significantly reduced the expression of A20 and Bcl-3; *S*. *thermophilus* TMC1543 and *B*. *bifidum* TMC3108 reduced levels of Bcl-3 and; *B*. *bifidum* TMC3115 reduced Bcl-3 and MKP-1 (Fig. 8). On the contrary, *L*. *rhamnosus* LA-2 significantly increased levels of A20 and MKP-1 in adipocytes while *L*. *paracasei* TMC0409 increased A20 and Bcl-3.

## Discussion

From the histological point of view, adipose tissue is composed of adipocytes (mature fat cells) and the interadipocytar stromal-vascular fraction formed by extracellular matrix with dispersed fibroblasts, preadipocytes, endothelial, and immune cells [27]. Excessive growth of adipose tissue in obesity is the result from enlargement of existing adipocytes (hypertrophy) and formation of new adipocytes (hyperplasia) through differentiation of stromal preadipocytes (adipogenesis) [28]. Mature adipocytes represent 50–85% of the total cellular components of adipose tissue. Obese subjects are characterized by a higher total adipocyte number than lean individuals [29]. Moreover, the hypertrophic adipocytes in obese individuals shift their immune balance towards the production of pro-inflammatory molecules [30, 31]. Accordingly, microarray profiling of isolated adipocytes from obese *versus* non-obese Pima Indians revealed an increased expression of inflammation-related genes in obese adipocytes [32].

In this work, the expression profile of immune receptors and pro-inflammatory cytokines were examined in porcine preadipocytes (PIP cells) and differentiated adipocytes. TLRs that are usually expressed immune cells, but their expression has been also documented in non-immune cells like intestinal epithelial cells [23, 24, 26]. The expression of TLRs was also observed in adipose tissue [33], although this expression was mainly attributed to infiltrated macrophages [34]. However, murine derived preadipocyte and differentiated adipocyte cell lines (3T3-L1) have been shown to express TLRs in response to TLR ligands [33, 35]. Khazen et al. [36] reported an augmented expression of TLRs when 3T3-F442A cells were differentiated into adipocytes. TLRs are also expressed and are functional in human adipose tissue [37]. That work demonstrated that TLR2 and TLR4 were expressed at relatively high levels (compared to a monocyte cell line) on the surface of human adipose cells. Moreover, stimulation of human adipocytes with LPS, or with lipoteichoic acid, two specific ligands of TLR4 and TLR2, respectively, induced a strong increase in TNF-α production. Our results are in line with these previous observations since mRNAs from TLR1 to TLR9 were detected in both porcine adipose cells. Some preliminary experiments with TLRs agonists showed that these receptors are functional in porcine adipocytes, however further experiments are needed to fully evaluate TLRs signaling pathways in PIP cells and mature adipocytes.

Our work also demonstrated the expression of TNF-α receptors (TNFR-1 and TNFR-2) in both PIP cells and mature porcine adipocytes. Since the first description of enhanced expression and secretion of TNF-α by adipose tissue of obese rodents that linked inflammation to obesity and insulin resistance [38], several works have clearly demonstrated that TNF-α has a crucial role in the pathogenesis of inflammation and insulin resistance by promoting immune and metabolic complications. It is known that most effects of TNF-α on adipose tissue are mediated by the TNFR-1 and subsequent activation of various transduction pathways [11]. Two transcription factor-signaling pathways have been linked to the pro-inflammatory effects of obesity and insulin resistance: the NF-κB (nuclear factor-κB) pathway and the c-Jun NH2-terminal kinase (JNK) pathway. It is believed that overproduction of TNF-α in obesity is an ultimate attempt to control adiposity, since increased TNF-α may help at limiting further weight gain through lipolysis and insulin resistance, impaired preadipocyte differentiation and increased adipocyte apoptosis [39]. However, these effects of TNF-α occur at the expense of worsening insulin resistance and inflammation. TNF-α produced by adipocytes further up-regulate pro-inflammatory adipokines and cytokines [40, 41]. Culturing human adipocytes in media previously conditioned by TNF-α resulted in pro-inflammatory adipokine overproduction. This was abrogated by immunoneutralization of TNF-α in these media, indicating that TNF-α is a crucial and proximal contributor to adipokine dysregulation in adipocytes [40]. Activation of TNFR-1 pathway in adipocytes increases the production of other inflammatory factors such as IL-6 and MCP-1. The *in vivo* release of IL-6 by whole-body adipose tissue could contribute to 15–35% of the systemic IL-6 in human and circulating levels and adipocytes production of IL-6 are increased in obesity [42]. Infiltration of adipose tissue by macrophages is an important event in the increased inflammatory process in obesity. MCP-1 and its receptor (CCR2) are required for macrophage infiltration into adipose tissue. MCP-1-or CCR2-deficient mice fed a high-fat diet exhibited fewer macrophages and a lower inflammatory gene profile in adipose tissue together with reduced insulin resistance [43]. Treatment of 3T3-L1 differentiated adipocytes with MCP-1 decreased insulin-stimulated glucose uptake and the expression of several adipogenic genes [44]. Also, they report that the capacity of TNF-α to increase pro-inflammatory factors in adipose cells. Similarly, both PIP cells and mature adipocytes showed significant up-regulation in the expressions of MCP-1, IL-1α, IL-1β, IL-6, and IL-8.

Additionally, stimulation of porcine mature adipocytes with TNF-α significantly changed the expression of negative regulators of the TNFR-1 pathway. The zinc-finger protein A20 is a key player in the negative feedback regulation of the NF-κB pathway in response to multiple stimuli. It is known that TNF-α dramatically increases A20 expression in all tissues, including adipose tissue [45]. Interestingly, it was reported that the NADPH oxidase Nox4 acts as a switch from insulin-induced proliferation to differentiation by controlling MKP-1 expression, which limits ERK1/2 signaling in human and mouse preadipocytes [46]. The work clearly showed that Nox4 controls the expression of MKP-1 and thereby limits the contribution of the proliferative Ras–Raf-ERK1/2 pathway to insulin signaling. ERK1/2 phosphorylates IRS-1 on serine-residues and thereby prevents IRS-1 tyrosine phosphorylation. The Nox4-dependent induction of MKP-1 prevents this effect and therefore promotes insulin-induced differentiation but attenuated insulin-induced proliferation. Those works demonstrated that negative regulators have important roles in the biology of adipocytes, with impacts not only in immune responses but in proliferation and differentiation as well.

The data presented in this work showed that our porcine *in vitro* systems (PIP cells and mature adipocytes) share all the immunological characteristics that have been attributed to these cells in other species, especially human. Then PIP cells and the porcine mature adipocytes obtained from them, could be useful laboratory tools to gain insight into the immunobiology of adipose tissue, as well as for the screening and evaluation of potential therapies aimed to beneficially modulate adipose immune response. In relation to this last assumption, we demonstrated here that our porcine *in vitro* systems are of value for the evaluation of immunobiotic effects.

Recently, gut microbiota has been identified as an important modifier of systemic inflammatory reactions influencing remote tissues [47]. Interestingly, different gut microbiota-derived products can exert both pro- and anti-inflammatory effects. It was described that translocation of LPS and peptidoglycans from microbiota into systemic circulation leads to metabolic endotoxemia, suggested as one of the main triggers of adipose tissue and systemic low-grade inflammation [47, 48]. On the contrary, products of gut bacterial fermentation such as short-chain fatty acids (SCFA) were shown to have anti-inflammatory effects and influence energy homeostasis [49]. In addition, several works demonstrated that orally administered probiotics are able to modulate tissues distant from the gut including the respiratory tract [50], blood [51], bone marrow [52], and adipose tissue [17, 18]. These studies showed that in addition to translocated microbial products, immunobiotics are able to modulate distant tissues through their ability to modify cytokine’s profiles [50–52].

In this work we investigated the possibility that LAB modify the response of porcine adipocytes to TNF-α stimulation through host’ intestinal immune-competent cells. We treated the porcine immune cells from Peyer’s patches with different LAB strains and, tested conditioned media from LAB-stimulated immune cells to determine the regulatory effects on porcine preadipocytes (PIP cells) and differentiated adipocytes. As it is described for other probiotics’ effects, we found strain specific effects of LAB on PIP cells and differentiated adipocytes.

**Fig 9:**
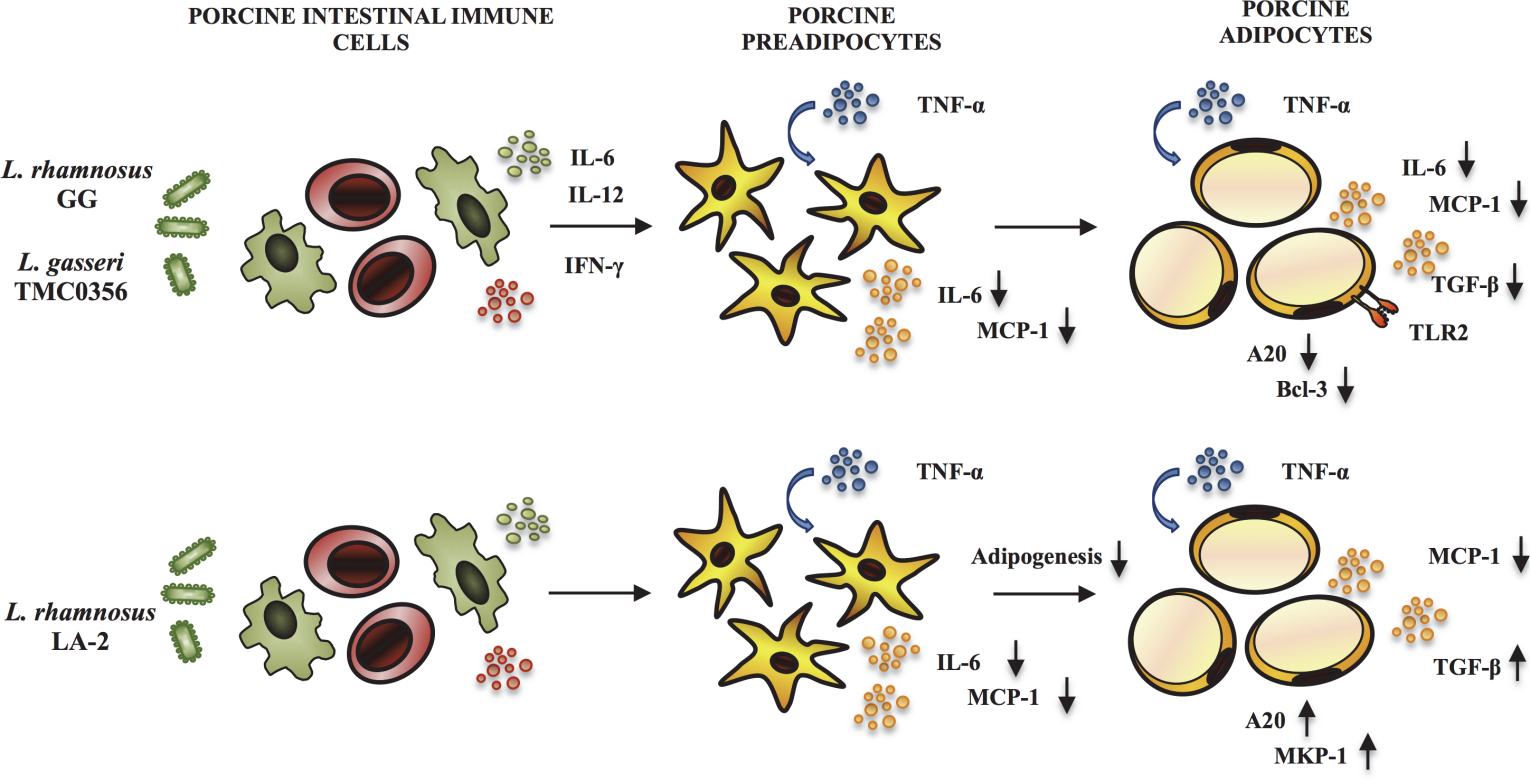
Possible immunomodulatory activity of *L*. *rhamnosus* GG, *L*. *gasseri* TMC0356, and *L*. *rhamnosus* LA-2 in both PIP cells and differentiated adipocytes after stimulation with TNF-α.

The *Lactobacillus* GG, *L*. *gasseri* TMC0356, and *L*. *rhmanosus* LA-2 showed remarkable effects with significant reduction in the expression of pro-inflammatory cytokines and chemokines in adipocytes challenged with TNF-α (Fig. 9). The strains *Lactobacillus* GG and *L*. *gasseri* TMC0356 reduced the expression of TLR2, A20 and Bcl-3, while A20, MKP-1 and TGF-β were up-regulated by *L*. *rhmanosus* LA-2 in adipocyte cells. The results of *Lactobacillus* GG or *L*. *gasseri* TMC0356 mediated down-regulation were expected since some previous publications documented the capacity of these two strains to influence adipocytes immunobiology. However, a mechanism behind the *L*. *rhmanosus* LA-2 mediated up-regulation was unknown. In addition, we previously used the conditioned medium of murine macrophage-like cell line J774.1 cultured with LGG or TMC0356 strains to stimulate mouse preadipocyte cell line 3T3-L1 and found a suppressed lipid accumulation and reduced PPAR-γ mRNA expression [19]. Moreover, the J774 cells treated with *Lactobacillus* GG or *L*. *gasseri* TMC0356 increased production of cytokines IL-6 and IL-1, suggesting that lactobacilli may suppress differentiation of preadipocytes through macrophage activation and production of Th1 cytokines. Several *in vivo* studies have comparatively evaluated the immunoregulatory effects of *L*. *gasseri* TMC0356 and *Lactobacillus* GG. Kawase et al. [53] demonstrated that oral administration of *Lactobacillus* GG or *L*. *gasseri* TMC0356 alleviate nasal allergic symptoms by suppressing the increase in nasal vascular permeability caused by local inflammation associated with allergic rhinitis in rodents. Moreover, in an allergic rhinitis guinea pig model, both LAB strains were able to decrease the total numbers of leukocytes, particularly eosinophils and neutrophils from the nasal cavity lavage fluid, and the OVA-specific IgE concentration in the serum [54]. *In vitro* studies of the immune responses of murine Peyer's patches stimulated with *Lactobacillus* GG or *L*. *gasseri* TMC0356 showed the capacity of both strains to increase the production of IL-6, IL-12 and IFN-γ by intestinal immune cells [55]. Those studies clearly indicate that both *Lactobacillus* GG and *L*. *gasseri* TMC0356 are equally effective in improving Th1 response not only in the gut by in the systemic compartment as well. Similarly, in this work, CFS from cultures of porcine Peyer's patches with GG or TMC0356 strains were able to functionally modulate the response of differentiated porcine adipocytes to TNF-α challenge. Then, our data suggest that Th1 cytokines produced by intestinal immune cells will be also capable of downregulating expression of pro-inflammatory genes in mature adipocytes (Fig. 9). In line with this assumption, *L*. *gasseri* TMC0356 was found to be able to stimulate the respiratory immune responses in a diet-induced obese mouse model, indicating that this immunobiotic strain may protect host animals from the lung immune dysfunction caused by obesity [56].

## Concluding Remarks

Taking into consideration that several *in vivo* and *in vitro* studies clearly demonstrated the beneficial effects of *Lactobacillus* GG and *L*. *gasseri* TMC0356 in adipose inflammation, the results presented in this work indicate that the PIP cells and porcine adipocytes could be used for the screening and the selection of new immunobiotic strains with the potential to functionally modulate adipose inflammation when orally administered. Moreover, the results of this work demonstrated that the regulation of IL-6, MCP-1, A20 and Bcl-3 induction in TNF-α-challenged porcine adipocytes could be certain biomarkers to screen and select potential immunobiotic strains.
